# The association between neighbourhood-level deprivation and depression: evidence from the south african national income dynamics study

**DOI:** 10.1186/s12888-017-1561-2

**Published:** 2017-12-11

**Authors:** Nicholas Dowdall, Catherine L. Ward, Crick Lund

**Affiliations:** 1 Department of Social Policy and Intervention, University of Oxford, Oxford, UK; 2 Department of Psychology, University of Cape Town, Cape Town, South Africa; 3 Alan J Flisher Centre for Public Mental Health, Department of Psychiatry and Mental Health, University of Cape Town, Cape Town, South Africa; 4 Centre for Global Mental Health, Institute of Psychiatry, Psychology and Neuroscience, King’s College London, London, UK

**Keywords:** Depression, Neighbourhood, Deprivation, South Africa, CESD-10

## Abstract

**Background:**

Depression contributes substantially to the burden of disease in South Africa. Little is known about how neighbourhoods affect the mental health of the people living in them.

**Methods:**

Using nationally representative data (*N*=11,955) from the South African National Income Dynamics Study and the South African Indices of Multiple Deprivation (SAIMD) modelled at small-area level, this study tested associations between neighbourhood-level deprivation and depression, after controlling for individual-level covariates.

**Results:**

Results showed a significant positive association between neighbourhood-level deprivation and depression using the composite SAIMD (**β** = 0.31 (0.15); *p*=0.04) as well as the separate deprivation domains. Living environment deprivation (**β** =0.53 (0.16); *p*=0.001) and employment deprivation (**β** = 0.38 (0.13); *p*=0.004), respectively, were the two most salient domains in predicting this relationship.

**Conclusions:**

Findings supported the hypothesis that there is a positive association between living in a more deprived neighbourhood and depression, even after controlling for individual-level covariates. This study suggests that alleviating structural poverty could reduce the burden of depression in South Africa.

## Background

Depression contributes more to the global burden of mental and substance abuse disorders than any other single disorder [1] and projections are that it will be the second leading cause of disability in the world by 2020 [2]. There is a long tradition of inquiry from high-income countries (HIC) into the relationship between socioeconomic adversity and depression at the individual level. Variables such as low income [3], unemployment, low education, social class [4], and financial strain [5] all show associations with depression. Conversely, financial and physical assets have been shown to safeguard against common mental disorders such as depression [6]. More recently, evidence has begun to emerge on the nature of the ‘poverty–mental health’ relationship in low- and middle-income countries (LMIC) [7].

Recent studies have begun to use the neighbourhood, rather than the individual, as a unit of exposure. At neighbourhood level, a promising body of empirical literature has begun to show significant associations between specific neighbourhood characteristics and depressive symptoms across a number of countries and socioeconomic groups [8, 9]. For example, neighbourhood social disorder has been consistently found to be associated with depressive symptoms, and higher neighbourhood socioeconomic status (SES) to function as a protective factor against depression [10]. Low neighbourhood SES and poor social cohesion have been shown to be significantly and independently associated with poor mental health status in the UK, after adjusting for individual SES [11], while improvements in aspects such as community-based grassroots organisations and shared public spaces for recreation, have been associated with improved mental health outcomes in China [12].

Neighbourhood characteristics can therefore function as either stressors or buffers that either increase or decrease the likelihood of depression [13]. Features like lack of neighbourhood resources, violence or poor social cohesion could function as stressors, while physical and social characteristics of neighbourhoods like access to social support may function as buffers [14].

Yet almost all the studies in this field have been conducted in high income countries (HIC) [8, 10]. In one of the few studies of its kind in LMIC, Tomita and Burns [15] addressed this gap with a study exploring correlations between neighbourhood social capital and depression in South Africa, and found that perception of social trust and the extent to which participants ‘preferred’ living in their neighbourhoods were significantly negatively associated with depression. A second study by Tomita, Labys and Burns [16] found a positive and significant relationship between perceived neighbourhood social disorder (characterised by violent and threatening behaviour) and depressive symptomatology. However, both studies had a risk of same-source bias in that self-report data was used for both the outcome variable and the neighbourhood variable of interest.

South Africa presents a unique environment in which to further explore how neighbourhoods impact on mental health outcomes. Apartheid social planning not only conflated race and class but located inequality, poverty and exclusion geographically by neighbourhood, a legacy which persists [17]. Deprivation in South Africa shows clear patterns with regard to it’s geographic location of deprivation: for instance, former Black homeland areas still remain the most deprived regions [18].

This study therefore explores associations between neighbourhood-level deprivation and individual’s depressive symptoms in a nationally representative South African sample. It avoids the same-source bias problem by combining comprehensive individual-level depression and socioeconomic data with independently sourced neighbourhood-level deprivation data. This neighbourhood deprivation data can be analysed both as a composite index as well as along individual domains, allowing for a more focussed investigation of how specific area-level factors influence mental health.

## Methods

### Design and setting

Data collected as part of the National Income Dynamics Study (NIDS), commissioned by the South African Presidency in 2006 [19], was combined with the South African Indices of Multiple Deprivation (SAIMD) [20]. The NIDS data provides nationally representative data on expenditure, income, assets, access to services, education, employment, health and dimensions of well-being, including depression [19].

To obtain neighbourhood level data, we used one of the latest versions of the SAIMD, which have been modelled at datazone-level. “Datazones” are geographical units containing approximately 2000 inhabitants and describe “pockets of deprivation by maximising social homogeneity and population density homogeneity” [21]. The datazone represents a more specific and meaningful unit with which to analyse deprivation, compared to larger areas such as wards, districts or municipalities [20]. The variation present in such large areas limits the potential to comment on factors like neighbourhood-level deprivation [22]. This SAIMD data was merged with the NIDS data to enable an analysis of individuals ‘nested’ within neighbourhoods.

This study investigates the association between neighbourhood-level deprivation in 2007 and individual depression scores in 2008, with controls for other individual covariates included. It was hypothesised that high area-level deprivation (both composite and individual dimensions) would be positively associated with depressive symptoms after controlling for individual-level variables. No specific hypotheses were made with regards to which domains of deprivation would be the most salient predictors of individual depression.

Ethical approval for NIDS was granted by the University of Cape Town (UCT) Commerce Faculty Ethics Committee [19] and this study was approved by the Humanities Research Ethics Committee at UCT. The NIDS data collectors administered an informed consent process with all participants, and only proceeded with interviews once this process was complete and they were satisfied that the participant fully understood all aspects of the research.

### Participants

Private households from every province constituted the sample [19]. The spread of sampling units per province and per geography type closely mirrored a ‘master sample’ used by Statistics South Africa (the officially recognised body producing national statistics such as the census in South Africa) between 2004 and 2007 for various household surveys and was thus seen as satisfactorily representative in this regard [19].

Each household member aged 15 or older was requested to complete an adult questionnaire. Further, the oldest woman in each household or the next resident most knowledgeable about living arrangements completed a household questionnaire. Data were used from the individual and household questionnaires as well as the individual and household-derived variable files created by NIDS. Approximately 16,800 adults spread across 400 primary sampling units were sampled. The response rate for households was 69%, but once a household had been sampled, the individual response rate within that household was 93.3% [19]. There were some differential patterns of responses across the urban/rural divide, with the rural response rate at 76.5% and the urban response rate at 67.5%. Similarly, there were some differential patterns across ethnic groups, with Black African response rate at 76%, while the White^1^ response rate was substantially lower at only 36%, a pattern that has been well documented in South African survey research [23]. However, these figures are only estimates, as information on the racial profiles of non-responding households was not collected, so estimates are based on the predominant ethnic group per Primary Sampling Unit.

### Measures

#### Centre for epidemiologic studies short depression scale (CES-D 10)

The NIDS adult questionnaire includes the ten-item CES-D [24]. The scale was designed to measure depressive symptoms in the general population and is one of the five most commonly used self-report measures of depressive experiences [25]. The CES-D has good psychometric properties, displaying high convergent validity with clinical scales such as the Beck Depression Inventory (*r* = 0.81) and the Zung Self-Rating Depression Scale (*r* = 0.90) [25]. Responses are recorded on a 4-level Likert-type scale of frequency ranging from ‘rarely or none of the time’ to ‘all of the time’, with scores ranging from 0 to 30 and higher scores indicating greater depression. Cronbach’s alpha for this scale in this sample was 0.74. The CES-D 10 scores were summed in order to calculate a total score for depressive symptoms. Originally designed to measure depression symptoms in the general population, the CES-D 10 best conceptualises depression on a continuum ranging from emotional well-being to depression rather than a dichotomy [24, 25].

#### Area-level deprivation

The deprivation indices used in the SAIMD were constructed from Statistics SA 2007 Community Survey data, which sampled 274,348 dwelling units across all nine provinces in South Africa [20]. It is a nationally representative household survey designed to provide information on the population between censuses. The indices were constructed along four domains using eleven indicators. These deprivation domains are: income and material, employment, education, and living environment.

The indices provide an overall deprivation score for each datazone, ranging from 0 to 400, with higher scores indicating greater deprivation. The four domains were equally weighted in the construction of the overall deprivation score. Each individual dimension was given a score from 0 to 100. For example, a high-scoring neighbourhood on the Income and Material Deprivation domain is one where most residents are living on an income of less than 40% of the population mean income and lack durable goods such as refrigerators. Similarly, a high-scoring neighbourhood in the Living Environment domain is one in which most residents are living in shacks, or over crowded houses without piped water or electricity, for example. For a detailed breakdown of the indices and indicators see Noble and Wright [26]. The scores represent exponentially transformed domain ranks of the domain scores [20].

#### Individual-level covariates of depression

A comprehensive set of questions relating to socioeconomic, demographic and general health information was included in the NIDS questionnaires. Without proper consideration of relevant individual-level information in the analysis, neighbourhood-level variables are likely to act “partially or entirely as proxies for individual attributes and, as such, a partition of the contribution of each to the chosen health outcome of depression becomes impossible” [27] (p.116). As such, certain individual-level variables including age, gender, marital status, employment status, education level and income were considered for inclusion in the analysis. Two of the most consistent findings across the depression literature in developing and developed contexts are that depression follows a course that peaks in middle-age [28] and that females are at greater risk of depression than males [29, 30]. Being married or living with a partner can also function as a protective factor against common mental disorders [29]. Evidence from LMIC has shown education to be negatively and independently associated with depression [28, 31]. Employment status is widely viewed as an important covariate for common mental health disorders, with secure employment acting as a protective factor [32]. Though findings on the association between income and depression have been inconsistent, they have still shown significant associations in many studies, with low income representing a risk factor [32]. Negative life events such as the death or serious illness of a family member, being a victim of theft, or job loss, have been reported as predictors of common mental disorders [33]. The negative events variable was created from a checklist of 12 items, ranging from death or serious illness to job loss and destruction of household property. An interaction between negative life events and deprivation will be explored in order to see how deprivation salience varies in the presence and absence of negative life events.

Individual-level covariates were also constructed using the NIDS data, in order to mirror the SAIMD neighbourhood-level domains. A binary durable goods variable was constructed by ascertaining whether an individual lived in a house without a refrigerator, television or radio. Secondly, a binary individual-level living environment deprivation variable was calculated which measured whether people were living in houses without on-site running water, electricity for lighting, a toilet or pit latrine, or were living in shacks. These composite binary variables were included in models to bring about consistent matching of the individual-level variables to the neighbourhood-level deprivation variables.

#### Data analysis

All statistical analyses were carried out using the Stata 12 software package. GPS co-ordinates (geo-location) of each NIDS household were fitted to their respective datazone (and hence to the SAIMD neighbourhood deprivation score) via the polygon shape file provided by the Centre for the Analysis of South African Social Policy (CASASP) that specifies datazone boundaries. Individuals with missing GPS co-ordinates and CES-D10 scores were excluded from the sample (*N* = 206). This subsample did not differ in any significant way on the variables of interest from the rest of the sample. The sampling and post-stratified design weights recommended by NIDS were used in the analysis [34]. Both the composite SAIMD index and the domain scores were converted into z-scores in order to more meaningfully interpret their coefficients.

The ‘cluster’ corrections were applied in the weighting process, and this had a bearing on the types of models that could be used in the statistical analysis. When a sample design is two-stage, a Primary Sampling Unit (PSU) or ‘cluster’ is initially sampled and then units of households and individuals are sub-sampled from within the cluster. The assumption of simple random sampling ignores the fact that two people within the same cluster or PSU are likely to be more similar than two people chosen at random from the population due to what can be referred to as a ‘cluster effect’ [34]. If standard errors are not corrected for cluster effects, the cluster effects are more likely to produce significant associations, but these would be premised by the assumption that they do not exist in the data. This is very seldom true [34]. There are various reasons why these effects might exist. For example, people within neighbourhoods often have the same infrastructure and access to resources. Similarly, neighbourhoods often share common features relating to language, culture, and attitudes [34]. Certainly, in a context like South Africa with its long history of geographically structured oppression, it is important to consider cluster effects in the data.

To estimate cluster-robust standard errors in the presence of nested multi-level clustering, the svy set of commands in Stata were used. This was done to apply the NIDS post-stratification weights and cluster corrections to the sample. This introduced into the models a comparable measure of control to that which area-level specific and individual-level specific random effects on the intercepts would have achieved, particularly given that there were a large number (*n* = 417) of PSU’s [35]. As such, survey regressions were conducted for all models.

A two-stage process was used to test each of the hypotheses, using Ordinary Least Squares survey regression models. First, bivariate correlations between depression and each neighbourhood-level variable were calculated. Second, models controlled for all specified individual-level covariates. Models were run for the composite SAIMD, as well as for each of the four domain scores. Results were considered significant at *p* < 0.05. Missing data was handled through listwise deletion of cases from models. An interaction between negative events and deprivation was explored to see how deprivation salience varies in the presence and absence of negative events.

## Results

For the adult sample of the first wave of NIDS, 12,448 individuals were successfully mapped onto 417 datazones and had completed the CES-D10 portion of the individual adult questionnaire. Of these individuals, 11,955 had all the necessary data for incorporation into the full models: see Tables 1 and 2 for a description of this sample.

**Table 1:** Descriptives: Categorical socio-demographic variables (*N* = 11,995)

Variable	
Female	7473 (62.5%)
Male	4482 (37.5%)
Race	
African	9494 (79.41%)
Coloured	1568 (13.12%)
Asian/Indian	163 (1.36%)
White	730 (6.11%)
Marital Status	
Married	3516 (29.41%)
Living with partner	1050 (8.78%)
Widow/widower	1011 (8.46%)
Divorced or separated	351 (2.94%)
Never married	6027 (50.41%)
Education	
Below Gr 9	5437 (45.48%)
Above Gr 9	6518 (54.52%)
Employment Status	
Not economically active	4888 (40.89%)
Unemployed discouraged	781 (6.53%)
Unemployed strict	1482 (12.40%)
Employed	4804 (40.18%)
Living Deprivation Status	
Not deprived	5526 (46.22%)
Deprived	6429 (53.78%)
Durable Goods	
No	5265 (44.04%)
Yes	6690 (55.96%)
Urban	
Rural/traditional	6096 (50.99%)
Urban	5859 (49.01%)
Negative Events	
None	9470 (79.37%)
One or more	2466 (20.63%)

**Table 2:** Descriptive statistics: Continuous Variables in sample *(N* = 11,955)

Variable	Actual Range	Possible Range	Mean (Std Dev.)	Median	IQR
CES-D10	0–30	0–30	8.01 (4.76)	7	5–11
SAIMD Composite Index	0.1186/s12888-017-1561-24.08–368.23	0–400	145.59 (85.61)	138.21	79.73–207.96
SAIMD Income and Material	2.5–99.41	0–100	77.30 (24.49)	87.99	67.67–94.18
SAIMD Employment	5.23–87.08	0–100	43.348(21.41)	44.68	25.05–61.05
SAIMD Education	4.00–77.87	0–100	33.68 (16.06)	34.02	22.31–44.74
SAIMD Living Environment	1.35–99.91	0–100	68.05 (32.81)	81.72	40.55–97.78
Age	15–101	–	37.58 (17.05)	35	23–50
Household Income	0–136,968.7	–	4791.78 (8146.99)	2327.71	1271.94–4837.72

*Note.* Monthly household Income figures in South African Rand

We found significant bivariate associations (*p* < 0.001) between the composite SAIMD and depression scores (see Table 3 and 4), as well as for all four individual domains. The positive coefficients indicate that individuals living in more deprived datazones reported significantly more symptoms of depression. The coefficients represent the change in the CES-D10 score per standard deviation increase in the deprivation variable.

**Table 3:** Weighted bivariate associations between 2008 CES-D10 scores and all area-level and individual-level independent variables

CES-D10	*Standardised* β	*SE*	*P > t*	*R* ^*2*^
SAIMD Composite Index	0.92	0.12	<0.0001	3.5%
SAIMD Income and Material	0.88	0.10	<0.0001	4.7%
SAIMD Employment	0.95	0.12	<0.0001	3.6%
SAIMD Education	0.83	0.14	<0.0001	2.8%
SAIMD Living Environment	0.91	0.12	<0.0001	4.1%
HH Income	−0.82	0.10	<0.0001	
	*Unstandardised* B			
Age	0.02	0.00	<0.0001	
Male	−0.91	0.13	<0.0001	
Race [African]				
Coloured	−1.57	0.38	<0.0001	
Asian/Indian	−2.01	0.85	0.018	
White	−3.20	−0.30	<0.0001	
Marital status [Married]				
Living with partner	1.50	0.26	<0.0001	
Widow/widower	2.36	0.28	<0.0001	
Divorced/separated	1.56	0.59	0.008	
Never married	0.72	0.18	<0.0001	
Gr 9 or more education	−1.81	−0.18	<0.0001	
Durable goods	−1.50	0.19	<0.0001	
Living deprived	1.28	0.25	<0.0001	
Urban	0.80	0.26	0.002	
Employment status [Employed]				
Not economically active	0.77	0.16	<0.0001	
Unemployed discouraged	0.90	0.31	0.004	
Unemployed strict	1.61	0.24	<0.0001	
Negative life events reported	0.09	0.21	0.677	

Note. A higher CES-D10 score represents more depressive symptoms, therefore a positive coefficient implies more depressive symptoms and a negative coefficient fewer depressive symptoms

Square brackets indicate reference group for categorical variables

**Table 4:** *Beta coefficients from linear regression models for 2008 CES-D10 depression scores across deprivation domains (N = 11,955)*

CESD10	SAIMD Composite Index	SAIMD Income and Material	SAIMD Employment	SAIMD Education	SAIMD Living Environment
Deprivation (Standardised β)	0.31 (0.15)*	0.36 (0.16)*	0.38 (0.13)**	0.28 (0.15)	0.53 (0.16)**
Age	0.03 (0.01)***	0.03 (0.01)***	0.03 (0.01)***	0.03 (0.01)***	0.03 (0.01)***
Male	−0.69 (0.13)***	−0.68 (0.13)***	−0.68 (0.13)***	−0.7 (0.13)***	−0.66 (0.13)***
Marital status [married]					
Living with partner	0.85 (0.26)**	0.8 (0.26)**	0.84 (0.26)**	0.82 (0.26)**	0.8 (0.26)**
Widow/widower	0.88 (0.28)**	0.87 (0.28)**	0.86 (0.28)**	0.89 (0.28)**	0.88 (0.28)**
Divorced/separated	1.8 (0.52)***	1.78 (0.53)***	1.76 (0.52)***	1.82 (0.52)***	1.79 (0.52)***
Never married	0.48 (0.2)*	0.45 (0.2)*	0.44 (0.19)*	0.47 (0.2)*	0.44 (0.2)*
Race [African]					
Coloured	−1.23 (0.34)***	−1.13 (0.32)***	−1.15 (0.34)***	−1.32 (0.34)***	−1.11 (0.32)***
Asian/Indian	−0.4 (0.93)	−0.36 (0.9)	−0.33 (0.94)	−0.48 (0.91)	−0.27 (0.91)
White	−2.16 (0.39)***	−1.95 (0.4)***	−2.03 (0.4)***	−2.18 (0.39)***	−1.93 (0.38)***
Gr. 9 or more education	−0.95 (0.16)***	−0.94 (0.16)***	−0.97 (0.16)***	−0.91 (0.16)***	−0.95 (0.16)***
Employment status [employed]					
Not economically active	0.19 (0.17)	0.19 (0.17)	0.16 (0.17)	0.23 (0.17)	0.2 (0.18)
Unemployed discouraged	0.34 (0.32)	0.33 (0.32)	0.31 (0.32)	0.37 (0.32)	0.34 (0.32)
Unemployed strict	1.11 (0.21)***	1.09 (0.21)***	1.07 (0.21)***	1.15 (0.21)***	1.08 (0.21)***
HH Income (Standardised β)	−0.25 (0.12)*	−0.22 (0.12)	−0.24 (0.12)*	−0.24 (0.12)*	−0.23 (0.12)
Durable goods	−0.61 (0.19)**	−0.62 (0.18)***	−0.68 (0.19)***	−0.61 (0.18)**	−0.61 (0.18)**
Living environment deprived	0.16 (0.3)	0.16 (0.3)	0.17 (0.29)	0.2 (0.29)	−0.01 (0.3)
Urban	0.55 (0.27)*	0.52 (0.26)*	0.44 (0.25)	0.57 (0.26)*	0.73 (0.28)**
Negative events	0.14 (0.17)	0.14 (0.17)	0.16 (0.16)	0.17 (0.18)	0.2 (0.17)
Negative events x deprivation	−0.35 (0.2)	−0.41 (0.2)*	−0.43 (0.21)*	−0.21 (0.2)	−0.3 (0.19)
Constant	7.29 (0.43)***	7.29 (0.43)***	7.44 (0.42)***	7.24 (0.44)***	7.22 (0.44)***

Note. *p < .05. **p < .01.***p < .001

Square brackets indicate reference group for categorical variables

In terms of individual-level variables, a 1.00 standard deviation increase in household income elicits a 0.82 decrease in depression score. In the sample, being male is significantly associated with fewer depressive symptoms and a similar relationship holds for being younger. Coloured, Asian/Indian and White participants reported significantly fewer depressive symptoms when compared with Black African participants. Having been educated up to at least grade 9 and not being deprived of durable goods or in the living environment also functioned as protective factors against depressive symptoms. People living in urban environments displayed significantly more depressive symptoms than those who live in rural areas. Employed participants fared better than those in all the other employment categories. Those who had actively sought work without success showed significantly more depressive symptoms than the non-economically active participants. The category ‘unemployed strict’ denotes individuals who have actively sought out employment opportunities in the past four weeks, while ‘unemployed discouraged’ represents individuals who would like to have worked in the past month but have not actively searched for a job during that period. There is no evidence of an association between negative life events and reported depressive symptoms in the sample.

The full model for the composite deprivation index was significant: *F*(20,345) = 20.70, *p* < 0.0001 and had an *R* ^2^ = 0.113. The composite multiple deprivation coefficient’s significance remained at the 5% level after the inclusion of all the individual-level covariates *B* = 0.31 (0.15), *p* = 0.042. This indicates that a standard deviation increase in neighbourhood deprivation is associated with a 0.31 standard deviation increase in depression score, or approximately 1.5 points on the CESD-10 scale. Overall, the coefficients for the remaining explanatory variables remained quite similar across all the domains and resembled the original model closely. Of the four domains, Living Environment Deprivation was the most salient: *B* = 0.53 (0.16), *p* = 0.001. The Employment Deprivation domain was also a strong predictor of depression: *B* = 0.38(0.13), *p* = 0.004, while the Income and Material Deprivation domain was below the 5% level of significance: *B* = 0.35(0.16), *p* = 0.02. The Education Deprivation domain was the only non-significant area-level explanatory variables *B* = 0.28(0.15), *p* = 0.07.

The relationship between neighbourhood-level deprivation and depression is significant (higher deprivation leads to increases in depression scores for the composite and individual indices of deprivation), even after controlling for negative life events and the interaction of negative life events with deprivation. Reporting negative life events does not have an independent significant effect on depression scores for all indices of deprivation. For the composite index (as well as the Education and Living Environment indicies), the interaction of negative events with deprivation is also not a significant predictor of higher depression scores. That is, the effect of deprivation on depression scores is not mediated by the presence or absence of reporting a negative event. However, if we focus on the Income and Material, and the Employment indices respectively, the significant negative coefficient on the interaction between deprivation and negative events shows that for individuals that report negative life events (such as a death in the family), the effect of deprivation on depression is cancelled out.

## Discussion

Results of this study suggest that individuals living in more deprived neighbourhoods in South Africa experience more depressive symptoms than those living in less deprived neighbourhoods and this effect persists after controlling for a number of individual-level covariates of depression. Over and above individual-level factors, mechanisms operating at the neighbourhood-level appear to affect residents’ depression levels. These could take the form of place-bound effects, effects of shared social backgrounds, or peer effects, to name a few [22, 34].

Results from the income and material deprivation domain suggest that as the proportion of households with very low monthly incomes and few durable goods increases in neighbourhoods, individuals living in these neighbourhoods display more depressive symptoms. The aggregated effect of a community’s financial impoverishment has implications beyond those of individual circumstance. Future research should explore possible mechanisms whereby neighbourhood effects filter down to an individual level in the form of influential stressors or buffers that can affect mental well-being. Possibilities include neighbourhood quality and access to social resources [36]. Such factors are more likely to be salient in neighbourhoods with high levels of poverty [37] and, hence, residents experience unfulfilled needs and dissatisfaction that may be risk factors for depression. In situations of high material deprivation, cognitions relating to hopelessness, loss of control and helplessness have been linked to depression outcomes [38].

The employment domain was the second most salient in the ‘effect’ it exerted on depression symptoms. Experiments in social psychology have shown that prolonged deprivation along with basic needs not being satisfied can lead to feelings of incompetence and inefficiency as well as powerlessness and helplessness [39]. This in turn creates a perception of loss of control over one’s circumstances and often a sense of hopelessness [39]. The concomitant effects of anxiety and relative helplessness on mental health outcomes in such milieus are well documented [13, 40]. Another obvious outcome of highly concentrated levels of unemployment is that this facilitates delinquency, deviant peer affiliation (particularly among adolescents) and crime of various forms [37]. In South Africa, areas characterised by high rates of economic exclusion and poverty, where prospects of upward mobility are small, are highly susceptible to gang-related activity [41]. Reasons of despondency and disempowerment might also be help explain the association between unemployment and depression.

At an individual level, education has been consistently found to be a strong protective factor against depression [28, 31]. However the results indicated that although it was significant at the individual level, it was not significant at the neighbourhood level. Ross [13] reported a similar finding. This may be because individual-level education has more proximal and direct effects than the more distant neighbourhood-level education. The SAIMD definition of education deprivation is also a relatively crude measure and thus may be less sensitive to associations with depression.

For the composite index (as well as the Education and Living Environment domains), the interaction of negative events with deprivation does not have a significant effect on depression scores. That is, the effect of deprivation on depression scores is not mediated by the presence or absence of reporting a negative event. There were two exceptions to this result. For the Income and Material, and the Employment indices respectively, the significant negative coefficient on the interaction between deprivation and negative events shows that for individuals that report negative life events (such as a death in the family), the positive effect of deprivation on depression scores is cancelled out. A possible explanation for this could be that on the particular dimensions of income and material and the employment indexes of deprivation, the effect of neighbourhood deprivation on depression is attenuated in the presence of negative life events.

Various mechanisms could account for why neighbourhood level deprivation affects individual depression outcomes. It is likely that where a large percentage of houses lack basic amenities such as running water, toilets and electricity, this may serve as a proxy for deprivation of other resources and facilities within these communities. The more direct effect of this could relate to feelings of heightened insecurity, danger and humiliation among community residents. In areas with high population density and lack of resources such as toilets in houses and adequate lighting, the living environment lends itself to crime and violence [42]. Robins [43] highlights the “inextricably intertwined sanitation, safety and dignity issues” (p.494) that confront residents in a peri-urban settlement near Cape Town daily. Such characteristics are prevalent in many South African townships where levels of violent crime are very high [41]. Other possible ameliorative factors like street lighting or proximity to public services such as police stations or health care facilities are also likely to be lacking in these neighbourhoods. Evidence from studies in the Northern Cape Province of South Africa indicated that women viewed having electricity and lighting in their neighbourhood as the most important factor in improving their living conditions as it reduced their susceptibility to crime, physical violence and sexual assault [42, 44]. The appearance of a neighbourhood that constantly reminds its residents of pervasive poverty and a threatening environment is also likely to cause stress in the individuals living there and contribute to mental ill-health [37].

There is a substantial body of sociological literature and theorising which may assist in elucidating some of the social processes and mechanisms that affect health outcomes like depression. Sampson and colleagues [37] propose that two fundamental social processes operating at the neighbourhood level can be classified as ‘social capital’ and ‘norms and collective efficacy’. Social capital can be seen as consisting of cognitive, structural, bonding, bridging and linking mechanisms amongst social groups [36]. Norms and collective efficacy can be seen in the degree of mutual trust in communities and the extent to which expectations about neighbourhoods are shared among residents [37].

In essence, social cohesion in neighbourhoods regulates levels of informal social control and solidarity. High levels of neighbourhood social capital and its cognitive component, social cohesion, have consistently shown protective qualities for a variety of health outcomes, including common mental disorders and depression [13, 45]. However, the results of the present study may indicate that concentrated levels of deprivation, disadvantage and economic exclusion make it very difficult to foster the social capital, cohesion and collective efficacy that can protect residents from the stressors associated with physical and mental ill-health [37]. This is in line with the ‘social causation’ hypothesis which predicts that the accrual of multiple risk factors like lack of education, stress and lack of access to health care will increase vulnerability and could precipitate a mental illness like depression [46].

### Limitations

First, it must be acknowledged that this work refers to symptoms of depression rather than a formal diagnosis.

A second limitation is the lack of inclusion of a ‘neighbourhood tenure’ variable. Regrettably NIDS did not include a direct question about how long residents had been living in their particular neighbourhood. Without a tenure variable it is not possible to investigate cumulative exposures and lagged effects [22].

Third, a cross-sectional analysis limits any discussion of causality. This area of research would benefit greatly from longitudinal studies that can address same-source bias, reverse causation issues and cumulative effects [8]. Notwithstanding this limitation, it seems more likely that area-level deprivation leads to depression rather than the other way round, as it is unlikely that depressed individuals would drift into deprived areas on such a scale.

Fourth, the statistical method chosen used cluster corrected standard errors and survey regressions but was not a multilevel model. It was judged that this was an appropriate approach given the clustered nature of the data and the large number of primary sampling units (*n* = 417). However, the strength of multilevel modelling is its ability to examine both the role of individual-level and group-level predictors on individual-level outcomes simultaneously [47], and its use should be explored in future studies.

Fifth, while the datazone geographical units are by far the closest approximations of neighbourhoods available in South Africa, they are still slightly larger than equivalent units that have been used in other contexts [48]. As such, we should be mindful of problems such as ecological fallacy [49] and the modifiable areal unit problem [50]. For example, it is possible that, especially in more rural areas, the superimposed datazones were not able to sufficiently capture the social homogeneity. Similarly, inferences about individuals should not be drawn from this analysis, as it looks at the effect of group-level characteristics on individual outcomes.

Sixth, the lower response rates from urban, and white households must be acknowledged. While these trends are commonly observed in South African survey research [23], there is a possibility that the group that did answer, for example the 36% of white participants, systematically differed in some way from general population of white South Africans.

Finally, it must be acknowledged that even though multiple deprivation indices represent a far more sound proxy for relevant neighbourhood-level constructs than simple aggregated income proxies, they remain rather limited substitutes for the actual features of neighbourhoods, both physical and social, that are assumed to influence health outcomes [22].

### Future directions for research

A natural progression that has emerged from this particular study is to explore whether social capital and, more specifically, social cohesion modifies the association between neighbourhood deprivation and mental health. Theory and evidence suggest that socially cohesive neighbourhoods facilitate informal social control, which improves well-being of residents [37]. There is also evidence that mental ill-health is associated with area-level deprivation, but that this relationship is modified in cases of high social cohesion in neighbourhoods [11]. Recently, research using NIDS data found significant negative associations between neighbourhood social capital and depression [15]. Following on from the significant association found between neighbourhood-level deprivation and depression in this study, it seems logical to explore how neighbourhood social capital and deprivation interact in relation to depression.

### Implications for practice

Various forms of deprivation clearly have effects on mental health outcomes. Policy makers should incorporate this understanding into their intervention strategies. Strategies that have not traditionally been considered relevant for mental health, like housing and urban planning, are likely to have important mental health outcomes at a population level. Neighbourhoods are the units in which these strategies become actualised, in terms of the roll-out of integrated policies [22]. This study indicates that providing neighbourhoods with amenities such as adequate street lighting, water, sanitation, electricity and access to public transport, as well as addressing neighbourhood level material deprivation and unemployment could have beneficial implications for promoting mental health.

## Conclusion

This is the first empirical investigation into the relationship between depression and independently sourced neighbourhood characteristics in an African country and is one of very few in LMICs. The results support the hypothesis that there is a significant association between living in a more deprived neighbourhood and depression of the residents, even after individual-level covariates have been controlled for. This has demonstrated the existence of a key relationship for public health research and should be seen as a precursor to further investigation into the specific neighbourhood mechanisms that drive this process, such as social cohesion, physical resources and structures, or collective efficacy. Within the purview of research into poverty and mental health, this study illustrates another benefit of structural poverty alleviation and effective service delivery to policy makers.

## Abbreviations

CASASP: Centre for the Analysis of South African Social Policy
CES-D 10: Centre for Epidemiologic Studies Short Depression Scale
HIC: High-income Countries
LMIC: Low and Middle-income Countries
NIDS: National Income Dynamics Study
PSU: Primary Sampling Unit
SAIMD: South African Indices of Multiple Deprivation
SES: Socioeconomic Status
UCT: University of Cape Town

## Footnotes

1 The apartheid categories still influence many outcomes for South Africans (Coovadia, Jewkes, etc. 2009). Their use does not imply that the authors condone them: we do not.
